# Cardiac tissue engineering: Multiple approaches and potential applications

**DOI:** 10.3389/fbioe.2022.980393

**Published:** 2022-10-03

**Authors:** Ilaria Gisone, Antonella Cecchettini, Elisa Ceccherini, Elisa Persiani, Maria Aurora Morales, Federico Vozzi

**Affiliations:** ^1^ Institute of Clinical Physiology, National Research Council (CNR), Pisa, Italy; ^2^ Department of Clinical and Experimental Medicine, University of Pisa, Pisa, Italy

**Keywords:** cardiac tissue, scaffold, hiPSC, biomaterial, organoids

## Abstract

The overall increase in cardiovascular diseases and, specifically, the ever-rising exposure to cardiotoxic compounds has greatly increased *in vivo* animal testing; however, mainly due to ethical concerns related to experimental animal models, there is a strong interest in new *in vitro* models focused on the human heart. In recent years, human pluripotent stem cells-derived cardiomyocytes (hiPSC-CMs) emerged as reference cell systems for cardiac studies due to their biological similarity to primary CMs, the flexibility in cell culture protocols, and the capability to be amplified several times. Furthermore, the ability to be genetically reprogrammed makes patient-derived hiPSCs, a source for studies on personalized medicine. In this mini-review, the different models used for *in vitro* cardiac studies will be described, and their pros and cons analyzed to help researchers choose the best fitting model for their studies. Particular attention will be paid to hiPSC-CMs and three-dimensional (3D) systems since they can mimic the cytoarchitecture of the human heart, reproducing its morphological, biochemical, and mechanical features. The advantages of 3D *in vitro* heart models compared to traditional 2D cell cultures will be discussed, and the differences between scaffold-free and scaffold-based systems will also be spotlighted.

## Introduction

Cardiovascular diseases are a group of disorders (including coronary heart disease, stroke, hypertension, and congestive heart failure) (Burnett et al., 2021) that affect the structure and function of the heart and blood vessels and are estimated as the leading cause of death globally (WHO 2021). About 30% of worldwide deaths are related to cardiovascular disease, and, in industrialized countries, heart failure affects about 1–2% of the adult population (Miller et al., 2021; Camman et al., 2022). Besides lifestyle habits (smoking, obesity, lack of exercise) and hereditary factors, exposure to environmental pollutants represents an important cardiac risk factor, as confirmed by different studies (Shi et al., 2020; Burnett et al., 2021; Krishna et al., 2021). In this context, it is of utmost importance to have available, high-throughput and reliable systems to test as many substances as possible (Chaudhari et al., 2018; Burnett et al., 2021). For a long time, animals have been considered the gold-standard models for cardiotoxicity studies due to the limited availability of human cardiac cells. However, results obtained from animals cannot be directly translated to humans due to physiologic species-specific differences, especially in the maturation process of cardiomyocytes (CMs) and in the electrophysiological properties (Kaese and Verheule, 2012; Notbohm et al., 2019; Guo and Pu, 2020; Tsukamoto et al., 2020; Kofron et al., 2021; Yang et al., 2021). Moreover, animal testing presents ethical and moral aspects that need to be considered, and they are also expensive and need long-term housing. *In vitro* models have been developed to overcome these limitations, allowing the study of cellular response in a closed system, where the experimental conditions are maintained. Two-dimensional (2D) *in vitro* cell cultures have been widely used and have several advantages (Notbohm et al., 2019; Weng et al., 2020). However, due to the limitations associated with static cell cultures (Table 1) (Kapałczyńska et al., 2016; Tohyama et al., 2017; Ding et al., 2020), researchers recently focused on three-dimensional (3D) cultures, which provide a more reliable system to mimic the *in vivo* tissues environment and allow a more truthful approach to the study cell function and behavior. Thus, 3D cell cultures provide a good alternative for the *in vitro* imitation of human heart tissue. Generally divided into ***scaffold-based*** and ***scaffold-free***, 3D heart tissue models allow cells to organize themselves into a 3D structure resembling human myocardial cell organization. In Table 2, the main advantages of scaffold-free and scaffold-based systems are reported. Although 3D cultures are expensive and require specific and more complex technical skills than 2D cultures, they better replicate *in vitro* human cardiac tissue morphological, biochemical, and mechanical features*.* This mini-review intends to summarize scaffold-free and scaffold-based technologies available to provide heart tissues, suitable as *in vitro* models for disease investigation and treatment, drug and toxicological studies, and improve knowledge about CMs physiology.

**TABLE 1:** Comparison of pros and cons of 2D and 3D cell cultures.

Two-dimensional (2D) cell cultures	Three-dimensional (3D) cell cultures
Easiness	Replica of *in vivo* specific tissue environment
Reproducibility	Reliable tissue response
Low cost	Cell physiological function
Simple imaging and analysis	Preserved cell morphology, phenotype, and polarity
No specific technical skills are needed	
Limited cultivation surface area: low yield	Complexity
Unlike the *in vivo* environment, cells similarly exposed to nutrients, growth factors, and cytokines	High cost
Lack of cell-cell and cell-ECM interactions with effect on morphology, function, intracellular, organization, secretion pathway, and communication signaling	Bad reproducibility
	Specific technical skills needed
	More complex cell analysis due to the use of gels/scaffolds substrate (scaffold-based systems)

**TABLE 2:** Main advantages of scaffold-free and scaffold-based systems.

Scaffold-free	Scaffold-based
Easy to manufacture	Mimicking structural and mechanical properties of the tissue
Low number of cells	Improved cell adhesion, migration,differentiation, proliferation
Reduced time required for tissue construction	Provide bioactive cues to the cells for regulation for the activities
Self-organization of cells	Provide a physical environment for neovascularization and remodeling during the tissue development

## Cell types for *in vitro* studies

### Human primary cardiomyocytes

CMs are the leading cardiac cell component (70–80% of myocardial volume) responsible for heart function and contraction by coupling cytoplasmatic Ca^2+^ increase with force generation (Notbohm et al., 2019). CMs are connected between each other and with other different cardiac cell types, modulating the maturation of CMs as demonstrated by co-culture *in vitro* studies (Talman and Kivelä, 2018; Giacomelli et al., 2020; Tsukamoto et al., 2020; Campostrini et al., 2021; Takeda et al., 2021). The advantages of the primary CMs use are associated with the sarcomeric structure ideal for patch-clamp/contractility studies, the presence of ion channels ideal for Ca^2+^ imaging studies, a large number of available genetic models, and the responsiveness to hypertrophic stimuli (Peter et al., 2016). The main CMs limitation for *in vitro* studies is that they are non-dividing cells and have a limited lifespan (Onódi et al., 2022; Notbohm et al., 2019). In addition, during cell isolation, CMs lose the gap junctions, which can lead to cell death (Woodcock and Matkovich, 2005; Talman and Kivelä, 2018). Due to difficulties of working with adult CMs, many researchers turned to immortalized cardiac cell lines and human-induced pluripotent stem cell-derived CMs (hiPSC-CMs), which provide an excellent alternative to the direct use of human CMs.

### Immortalized cardiac cell lines: H9c2 and HL-1

H9c2 and HL-1 are immortalized cardiac cell lines derived from embryonic rat left ventricle and adult mouse atrium. HL-1 can proliferate and contract, while H9c2 are undifferentiated myoblast. These cells have simple culturing protocols and low cost maintenance (Kuznetsov et al., 2015). Comparative morphological analysis with primary cell cultures demonstrated that both H9c2 and HL-1 are characterized by a cardiac phenotype, also confirmed by mRNA expression studies. The transcriptomic analysis revealed the biochemical and bioenergetic similarity to primary CMs (Kuznetsov et al., 2015; Onódi et al., 2022). Considering all the limits related to the use of immortalized cell lines (first of all, the alterations of the cell cycle, which can lead to genetic and phenotypic alterations) (Jimenez-Tellez and Greenway, 2019), hiPSC-CMs have gained much more attention due to their human origin and a higher degree of similarity with human cardiac cells (Onódi et al., 2022).

### Human-induced pluripotent stem cells

hiPSCs, with their capabilities to differentiate in a wide spectrum of cell types, have revolutionized the world of basic and translational research. hiPSCs are obtained from somatic cells after the overexpression of four specific transcription factors (Oct4, Sox2, Kif-4, and c-Myc), and they can be expanded several times, maintaining their pluripotency (Camman et al., 2022). Compared to adult ventricular CMs, hiPSC-CMs show different morphology (round rather than rod-shaped) and metabolism (based on glucose rather than fatty acids), spontaneous beating contractions, limited myofibril alignment and sarcomere organization, lack of T-tubules, and alterations in excitation-contraction coupling. The cardiac markers analysis also confirmed immaturity (Denning et al., 2016; Silbernagel et al., 2020). The human heart is a complex organ containing different cell types, embedded in extracellular matrix (ECM) proteins, and exposed to electrical, mechanical, and biochemical stimuli. To overcome the limitations linked to immaturity, it is important to better reproduce the *in vivo* CMs microenvironment, with all the different stimuli to which cells are constantly exposed. For this reason, some researchers proposed the co-culture of hiPSC-CMs with other cardiac cell types (endothelial cells (ECs) and/or cardiac fibroblasts (CFs)) (Giacomelli et al., 2020; Zhao et al., 2020; Campostrini et al., 2021); others focused on long-time cultivation, the application of mechanical and electrical stimuli, addition of specific growth factors and specific adult-like metabolic substrates (insulin and fatty acids), and culture in 3D systems that provide important structural cues (Lundy et al., 2013; Camman et al., 2022).

## Three-dimensional *in vitro* cells cultures

Cells cultured in 3D can better recapitulate the *in vivo* specific tissue environment and its impact on cells, providing a more reliable tissue response (Cho et al., 2021). This is particularly true for the human heart, where the cells are organized into a versatile and dynamic network that 2D culture cannot replay (Giacomelli et al., 2017; Andrysiak et al., 2021). 3D *scaffold-free* cultures include systems obtained after self-aggregation of cells, while *scaffold-based* cultures enclose tissue replicas in which hydrogel tissue-engineered formats or scaffolds support cells. In both cases, cells achieve a 3D organization, which is important to preserve their morphology, phenotype, and polarity. Compared to 2D cultures, cells cultured in 3D show better viability and preserve physiological function (Kapałczyńska et al., 2016; Campbell et al., 2018; Andrysiak et al., 2021; Miller et al., 2021). However, 3D cultures still have some limitations since they are cost- and time-consuming and need specific technical skills. In addition, the non-human derivation of gels or scaffold substrates (e.g., Matrigel, polycaprolactone (PCL), hydroxybutyl chitosan (HBC)) in the scaffold-based systems may limit their clinical applications (Kapałczyńska et al., 2016; Sacchetto et al., 2020; Silbernagel et al., 2020; Caleffi et al., 2021; Campostrini et al., 2021; Cho et al., 2021; Camman et al., 2022). All these limitations explain the poor use of 3D cultures; nevertheless, in the last few years, the above described advantages have driven researchers towards more widespread exploitation of 3D systems.

## Heart models

**FIGURE 1:**
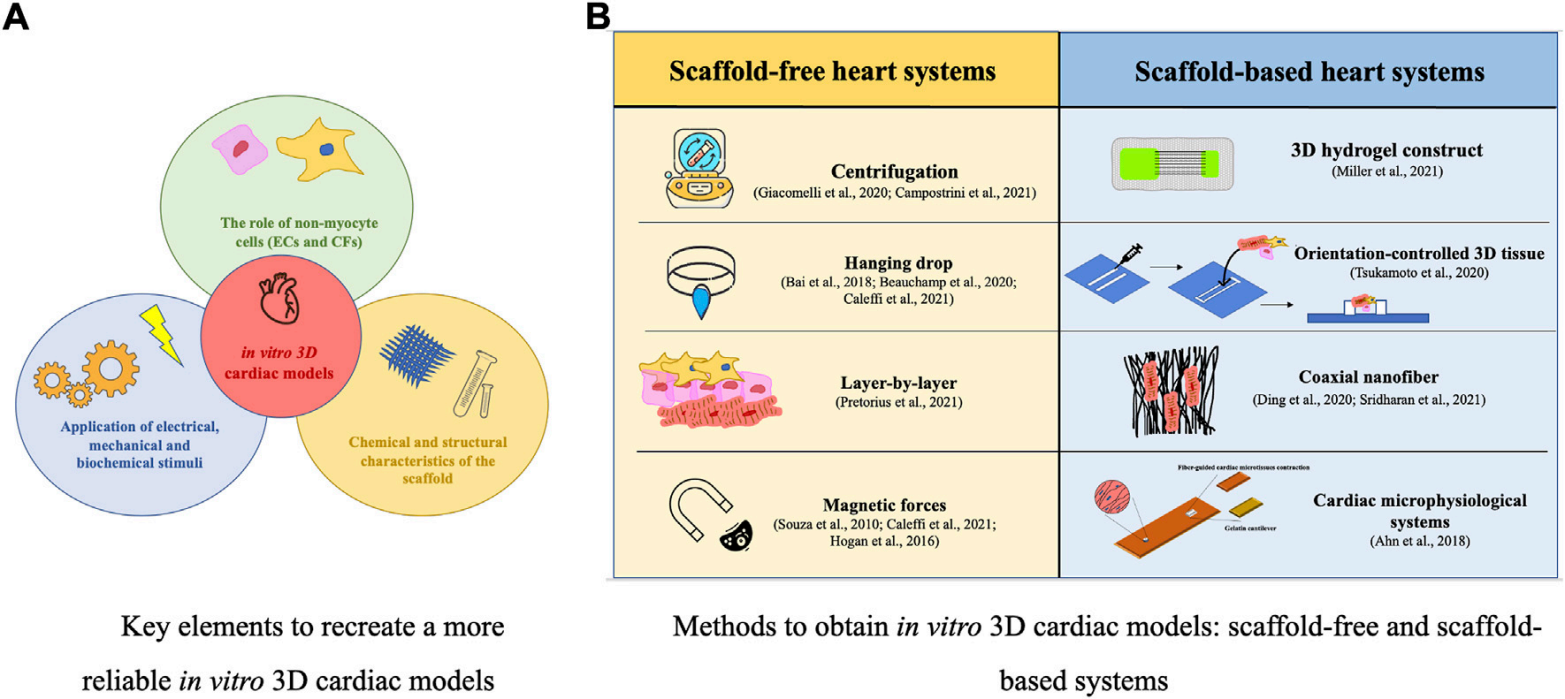
*In vitro* 3D cardiac models: key elements **(A)** and manufacturing methods **(B)**.

In the native myocardium, cells are embedded in a 3D ECM network, characterized by mechanical wholeness, that provides structural and biochemical signals involved in cell alignment and organization (Cho et al., 2021). 3D cardiac tissue systems mimic the heart ECM structure, supporting CMs and facilitating their cytoskeletal arrangement, morphology, gene expression, and function. (Beauchamp et al., 2020). Three key elements to recreate a more reliable *in vitro* 3D heart system should be taken into account (Sacchetto et al., 2020): 1) the role of non-myocyte cells (ECs and CFs), which are necessary for supporting the maturation and function of CMs, considering the specific ratio concentrations of different cells to better reproduce the *in vivo* microenvironment (Campostrini et al., 2021); 2) the application of electrical, mechanical, and biochemical stimuli (Kolanowski et al., 2020); 3) the chemical and structural characteristics of the scaffold (important in reproducing the architecture of the cardiac ECM, maintaining cells viability, providing the cell-cell and cell-ECM protein interactions and guiding CMs alignment) (Ding et al., 2020; Sridharan et al., 2021) (Figure 1A). Several methods have been recently proposed to produce 3D cardiac models, particularly spheroids or organoids, using different cell self-aggregation techniques (centrifugation, hanging-drop technology, layer-by-layer technique, magnetic systems), and biopolymeric printing devices to produce geometrically shaped scaffolds for cell homing (Figure 1B). The choice of the specific cardiac model is associated with the specific focus of the experiment as well as with the technical and economic availability of the laboratory. The 3D cardiac tissues described below are important *in vitro* tools for disease investigation, drug testing, and toxicological studies on cardiac cells. Furthermore, the use of hiPSCs, obtained from patients also opens the possibility of focusing on personalized medicine.

### Scaffold-free heart models

Scaffold-free systems can provide artificial 3D heart tissues without external support while maintaining mechanical integrity. *Spheroids* and *organoids,* generally defined as 3D microtissues (MTs), are examples. Both are miniaturized tissues able to reproduce the cell-cell and cell-ECM proteins interactions. Spheroids do not have limitations in the number/type of mature cells utilized, unlike organoids, which arise from tissue-specific stem cells or progenitor cells (harvested from different organs), have a more specific organ microarchitecture and more closely reproduce the functional tissue properties (Wang et al., 2021; Zarrintaj et al., 2022). MTs can be generated through centrifugation, hanging-drop, layer-by-layer, and magnetic forces techniques. MTs are easy and rapid to fabricate, need a low number of cells and can be composed of different cell types, improving the maturation of hiPSC-CMs at a structural, electrical, mechanical, and metabolic level. The scaffold-free systems facilitate morphological analysis since they are easily visualized with light, fluorescence, and confocal microscopy (Giacomelli et al., 2017, Bai et al., 2018; Beauchamp et al., 2020; Caleffi et al., 2021; Campostrini et al., 2021; Pretorius et al., 2021). Giacomelli et al. and Campostrini et al. proposed a scaffold-free spheroid model composed of CMs, ECs, and CFs (at a ratio of 70:15:15, respectively) that self-aggregate when subjected to centrifugation. The structural, metabolic, and functional maturation of hiPSC-CMs was confirmed through immunofluorescence staining of specific cardiac markers, video analysis of tissue contractions, action potential and RNA-seq analyses of sarcomere genes expression, and metabolic function investigations. The maturation features were achieved only in spheroids composed of three different cell types, indicating how important the cellular crosstalk is (Giacomelli et al., 2020; Campostrini et al., 2021).

In the hanging-drop technique, cell suspension droplets are posed at the bottom of a Petri dish lid, and due to gravity and surface tension, cells hang and self-aggregate. Bai et al. and Beauchamp et al. used this technique to produce spheroids by co-culturing hiPSC-CMs with other cardiac cell types. After confirming their beating contraction, researchers seeded spheroids into a mould cavity in continuous shaking to induce tissue compaction and maturation. At the end of the procedure, they obtained an intact cardiac patch characterized by synchronous beating activity (Bai et al., 2018; Beauchamp et al., 2020; Caleffi et al., 2021).

Petrorius et al. used the layer-by-layer technique to obtain an *in vitro* heart tissue model. After mixing hiPSC-CMs with the fibrin matrix, the solution was placed into a polycarbonate frame to produce the first layer on which the second ECs layer was deposited: the frame containing the cells was lifted off the dish surface and placed on polydimethylsiloxane supports. Thus, the tissue was entirely suspended in a fresh medium. The third CFs layer was eventually added. Immunofluorescent analyses of specific markers confirmed the migration and re-arrangement of the cells, with a stable expression level of particular genes (CD31, vimentin, α-SMA) (Pretorius et al., 2021).

In 2010, Souza et al. proposed magnetic force**s** to create 3D tissue models, exploiting the magnetic levitation of cells in a hydrogel consisting of gold, magnetic iron oxide nanoparticles, and filamentous bacteriophage (Souza et al., 2010). After incubation with magnetic nanoparticles (that electrostatically bind to the cell membrane), the cells are seeded in plates composed of non-adherent materials: using a specific magnet, the magnetized cells begin to levitate towards the air-liquid interface, aggregating and forming the 3D structure (Caleffi et al., 2021). Hogan et al. used this technique to provide a contracting 3D heart model, starting from cardiac cells obtained from neonatal rats (Hogan et al., 2016).

The disadvantages associated with self-aggregation techniques are related to the limits in the maturation level of CMs, different from that of CMs in the adult heart, (Campostrini et al., 2021) and in the heterogenous thickness of the cardiac system, due to difficulties in controlling the stacking of the cells (Bai et al., 2018). Considering the use of magnetic forces to aggregate the cells, although they provide the opportunity for precise temporal and spatial control of cells in an environment, the use of a powerful magnet may be responsible of the alteration of the cell behaviour and physiology (Souza et al., 2010; Caleffi et al., 2021).

### Scaffold-based heart models

Scaffold-based systems need specific support structures that guides the alignment of CMs, which is essential for their maturation (Miller et al., 2021). The cardiac tissue contractile force depends on layers of cells with different orientations. Consequently, it is vital to provide the support that can guide cell alignment, allowing the reproduction of heart-specific structure orientation. Scaffolds are porous structures composed of synthetic substrates and/or natural macromolecules that enable the supply of nutrients, growth factors, and gas exchanges. They facilitate cell adhesion, migration, proliferation, and differentiation. In some cases, before sowing onto 3D scaffolds, cells are mixed with specific polymers and/or hydrogel (Bai et al., 2018; Sacchetto et al., 2020; Tsukamoto et al., 2020; Caleffi et al., 2021; Miller et al., 2021). Based on the purpose of the experiment, it is possible to use scaffolds with different specific materials and properties. Miller et al. provided a micro-tissue using a 3D hydrogel construct as a scaffold, printed by a micro-continuous optical printing system that uses UV-light to polymerize a pre-polymer solution composed of cells (hiPSC-CMs and CFs) mixed with the gelatin methacrylate (GelMA). This solution was used to print the lines scaffold onto the pillar layer, composed of GelMA and marked with fluorescent beads. Beating contractions started in a few days, as seen through the displacement of the small, marked pillar layer. By tuning the light exposure time and the concentration of the GelMA, researchers can change the hydrogel pore size, controlling its stiffness. Analysis of stained sarcomere revealed a high degree of cell alignment, and gene expression analysis also demonstrated an increased expression of hiPSC-CMs’ maturity markers (CACNA1C, RYR2, MYH6, MYH7, MLC2C, TNNT genes) (Miller et al., 2021). Tsukamoto et al. proposed a method for manufacturing an orientation-controlled 3D heart tissue, combining different techniques. Using the HBC as ink material, the researchers fabricated a rectangular gel frame on a culture insert (3D print technology) to guide and control the orientation of the cells. They encapsulated hiPSC-CMs and normal human CFs into an ECM nanofilm (layer-by-layer technique) and then seeded them in the HBC frame (cell accumulation technique), able to guide cells’ orientation by limiting the direction of tension. The HBC gel is temperature sensitive, so it can be separated from the 3D cardiac tissue system. Using specific markers, the researchers analysed cell morphology and tissue contractile activity, demonstrating that cells contracted in the controlled 3D system in the same direction faster than cells cultured on uncontrolled tissue (Tsukamoto et al., 2020). Others have proposed aligned coaxial nanofiber as a scaffold to mimic the ECM heart structure to seed hiPSC-CMs. CMs aligned themselves with the nanofibrous structure and assumed an elongated morphology, resembling the *in vivo* morphology of adult CMs (Ding et al., 2020; Sridharan et al., 2021). The nanofibers may be natural materials (gelatin, collagen, fibrin) or synthetic polymers (PCL). A more efficient nanofibrous scaffold is made of PCL in the core and gelatin, a more adhesive material, as the coat. This system allows a high cellular adhesion and viability while maintaining the mechanical stretch property. The hiPSC-CMs alignment, in the same direction as the fibres, was confirmed by scanning electron microscopy (Sridharan et al., 2021). Ding et al. studied the morphology and differentiation stage of cardiac progenitor cells (hiPSC-CPCs) seeded on the nanofiber scaffold by fixing the contracting cells and staining them for TNNT and α-SMA. Results showed a higher expression of TNNT in cells cultured on a 3D scaffold than in 2D culture control, demonstrating a better maturation profile of hiPSC-CPCs, as confirmed by RT-PCR analysis. Moreover, synchronized intracellular Ca^2+^ oscillation and cell contraction analysis confirmed the improved differentiation and maturation profile of CMs cultured on a 3D-aligned nanofibrous scaffold (Ding et al., 2020). Ahn et al. printed nanofiber scaffolds (composed of PCL and dopamine hydrochloride), then functionalized with polydopamine (PDA) to facilitate cell adhesion. The microsystem is characterized by embedded sensors capable of providing information about the cell’s contractility. CMs from neonatal rats were seeded on the scaffold. Using the immunostaining technique, researchers evaluated the sarcomere Z-line alignment, length, and packing density, confirming the maturation profile of CMs. In addition, the Ca^2+^-sensitive dye revealed synchronous calcium transient, demonstrating the system’s ability to provide support on which CMs can mature into functional contractile tissue (Ahn et al., 2018).

The scaffold-based systems require high cost, specific technical skills, and long lead time for set up (Campostrini et al., 2021; Miller et al., 2021). Important technical disadvantages are related to the lack of cell-cell interactions (Caleffi et al., 2021) and to the cell distribution within the scaffold, which can be heterogenous, unlike the homogenous distribution *in vivo* (Campostrini et al., 2021). Furthermore, considering the aligned coaxial nanofiber scaffolds described above, a limit is related to the inclusion of only one cell type (CMs) (Ahn et al., 2018; Sridharan et al., 2021).

## Discussion

Thanks to their ability to better mimic the human cardiac tissue, in recent years, 3D cell cultures started to be used as models for drug and toxicological testing, providing an excellent alternative to *in vivo* animal studies, overcoming ethical concerns and problems related to species-specific differences with humans. Using hiPSCs as a source of cells to obtain CMs also opens the possibility of working with cells isolated from patients, thus paving the way for personalized medicine and disease investigation. Based on their objectives and knowing the specific advantages and disadvantages of each model, researchers can choose the more suitable *in vitro* 3D heart system. 3D systems can overcome the problem related to the maturation failure of *CMs in vitro*, and better results are obtained with co-culture with other cardiac cell types (endothelial cells, fibroblasts) (Giacomelli et al., 2017; Campostrini et al., 2021; Camman et al., 2022). Although 3D cardiac cultures can mimic the human heart tissue morphological, mechanical, and biochemical features (reproducing its cytoarchitecture), the reproducibility and accuracy of the model need to be improved. Despite some drawbacks, 3D heart systems could represent a significant step forward for research by recreating *in vitro* a reasonable substitute for the human heart on which different tests and analyses can be performed. Furthermore, considering the critical cell alignment obtained with the 3D scaffold-based systems, they could be used for regenerative procedures and pharmaceutical applications.

*In vitro* models can give information at molecular and cellular levels, but they are unable to provide insight on the physiological and systemic interactions between organs. For this reason, animals are still used as models and they are not yet replaceable in the study of multifactorial diseases (cardiovascular and neurodegenerative diseases, cancer) (Barré-Sinoussi and Montagutelli, 2015).

By integrating different *in vitro* 3D tissues, in the future it would be possible to partially re-produce the interconnection of the different organs, as in the human body, thus starting to over-come the *in vivo* animal testing.
